# Variation of pCO_2_ concentrations induced by tropical cyclones “Wind-Pump” in the middle-latitude surface oceans: A comparative study

**DOI:** 10.1371/journal.pone.0226189

**Published:** 2020-03-24

**Authors:** Haijun Ye, Evgeny Morozov, Danling Tang, Sufeng Wang, Yupeng Liu, Ying Li, Shilin Tang

**Affiliations:** 1 State Key Laboratory of Tropical Oceanography, Guangdong Key Laboratory of Ocean Remote Sensing, South China Sea Institute of Oceanology, Chinese Academy of Sciences, Guangzhou, China; 2 Southern Marine Science and Engineering Guangdong Laboratory (Guangzhou), Guangzhou, China; 3 Marine Hydrophysical Institute, Russian Academy of Sciences, Sevastopol, Russia; Universidade de Vigo, SPAIN

## Abstract

The Bermuda Testbed Mooring (BTM) and Bay of Bengal Ocean Acidification (BOBOA) mooring measurements were used to identify changes in the partial pressure of CO_2_ at the sea surface (pCO_2sea_) and air-sea CO_2_ fluxes (F_CO2_) associated with passage of two tropical cyclones (TCs), Florence and Hudhud. TC Florence passed about 165 km off the BTM mooring site with strong wind speeds of 24.8 m s^–1^ and translation speed of 7.23 m s^–1^. TC Hudhud passed about 178 km off the BOBOA mooring site with wind speeds of 14.0 m s^–1^ and translation speed of 2.58 m s^–1^. The present study examined the effect of temperature, salinity, dissolved inorganic carbon (DIC), total alkalinity (TA), air-sea CO_2_ flux, and phytoplankton chlorophyll a change on pCO_2sea_ as a response to TCs. Enhanced mixed layer depths were observed due to TCs-induced vertical mixing at both mooring sites. Decreased pCO_2sea_ (–15.16±5.60 μatm) at the BTM mooring site and enhanced pCO_2sea_ (14.81±7.03 μatm) at the BOBOA mooring site were observed after the passage of Florence and Hudhud, respectively. Both DIC and TA are strongly correlated with salinity in the upper layer of the isothermal layer depth (ILD). Strong (weak) vertical gradient in salinity is accompanied by strong (weak) vertical gradients in DIC and TA. Strong vertical salinity gradient in the upper layer of the ILD (0.031 psu m^–1^), that supply much salinity, dissolved inorganic carbon and total alkalinity from the thermocline was the cause of the increased pCO_2sea_ in the BOBOA mooring water. Weak vertical salinity gradient in the upper layer of the ILD (0.003 psu m^–1^) was responsible for decreasing pCO_2sea_ in the BTM mooring water. The results of this study showed that the vertical salinity gradient in the upper layer of the ILD is a good indicator of the pCO_2sea_ variation after the passages of TCs.

## Introduction

Anthropogenic CO_2_ emissions play an important role in global climate change. About 30% of the anthropogenic CO_2_ has been stored in the ocean [1]. The concentration of CO_2_ in oceanic water is under strong influence of the air-sea CO_2_ flux (F_CO2_) through the water surface [2]. The direction of CO_2_ gas exchange is governed by the difference between the partial pressure of CO_2_ in seawater (pCO_2sea_) and the atmosphere boundary layer (pCO_2air_), while the magnitude of CO_2_ gas exchange is mainly regulated by the sea surface wind speed [3]. In comparison with the small variation of pCO_2air_ [4], the variation of pCO_2sea_ plays a crucial role in determining the direction of CO_2_ gas exchange due to its sensitivity to episodic events, such as tropical cyclones (also called hurricanes and typhoons), vertical mixing, heavy precipitation, algae blooms, etc. [5–7].

Each year tropical cyclones (TCs) visit both the subtropical North Atlantic Ocean and the Bay of Bengal (BoB)–a semienclosed sea in the north Indian Ocean. TCs were found to have much influence on the marine environment, such as cooling of sea surface temperature (SST) [8–9], increase of dissolved oxygen concentration [10–11] and increase of chlorophyll a (Chla) concentration both at the surface and subsurface [9, 12–13] due to the ‘wind-pump’ effects induced by TCs [13–15]. The amplitude of the surface cooling strongly depends on the initial upper-ocean conditions such as mixed layer depth (MLD) and stratification in the thermocline along with intensity and translation speed of TC [8, 16–17]. Earlier studies using moored buoy measurements and model outputs have proven that the passage of a TC can cause strong positive value of F_CO2_ that is a net transfer of CO_2_ from the ocean to the atmosphere or efflux [5, 18–22]. However, most of the estimations of TC-induced F_CO2_ have been based on empirical assumptions: Bates et al. (1998) [5] assumed that the pCO_2sea_ remained constant during the TC and Perrie et al. (2004) [18] assumed that the pCO_2sea_ increased linearly and remained constant during the TC passage. Apparently, these assumptions were unreasonable because the variations of pCO_2sea_ should reflect the vertical mixing of the water column [21].

Distributions of pCO_2sea_ depend strongly on the temperature, dissolved inorganic carbon (DIC), total alkalinity (TA) and salinity [23]. Vertical mixing and upwelling associated with TC ‘wind-pump’ effects decrease the SST which in turn decrease the pCO_2sea_ by 0.0423°C^–1^ [23]. Such SST cooling was the dominant cause of the decrease of pCO_2sea_ during TCs Felix and Frances in the Atlantic Ocean [5, 21] and three TCs in the East China Sea [20]. On the other hand, TC-induced vertical mixing and upwelling can increase the pCO_2sea_ by the uplifting to the surface of deep water rich in DIC (i.e. CO_2_, ${HCO}_{3}^{-}$ and ${CO}_{3}^{2-}$). The increase of DIC was the dominant cause of the increase of pCO_2sea_ during TC Choi-Wan in the northwest Pacific Ocean [22] and tropical depression and TC Wutip in the South China Sea [6–7]. After the passage of typhoon Wutip in the South China Sea, the enhanced pCO_2sea_ was observed in the cyclonic eddy water due to the enriched DIC and decreased pCO_2sea_ was observed in the anticyclonic eddy water due to the decreased SST [7]. In addition to TC intensity and translation speed, the effect of a TC on pCO_2sea_ is determined by the vertical profiles of the ocean water properties [21].

To reveal the mechanisms responsible for the TC impact on the pCO_2sea_ in different water properties, the present study investigates the pCO_2sea_ response to TC Florence (September 2006) in the North Atlantic Ocean and Hudhud (October 2014) in the BoB using autonomous pCO_2_ observations. We summarize how the vertical salinity distributions affect the pCO_2sea_ in response to TCs. This study is a continuation of our previous study [14], and the first comparative study of TCs impact on the pCO_2sea_ variation in different water properties, which gives a consistent explanation for the pCO_2sea_ response to TCs in two cases.

## Data and methods

### Field measurements

**Fig 1:**
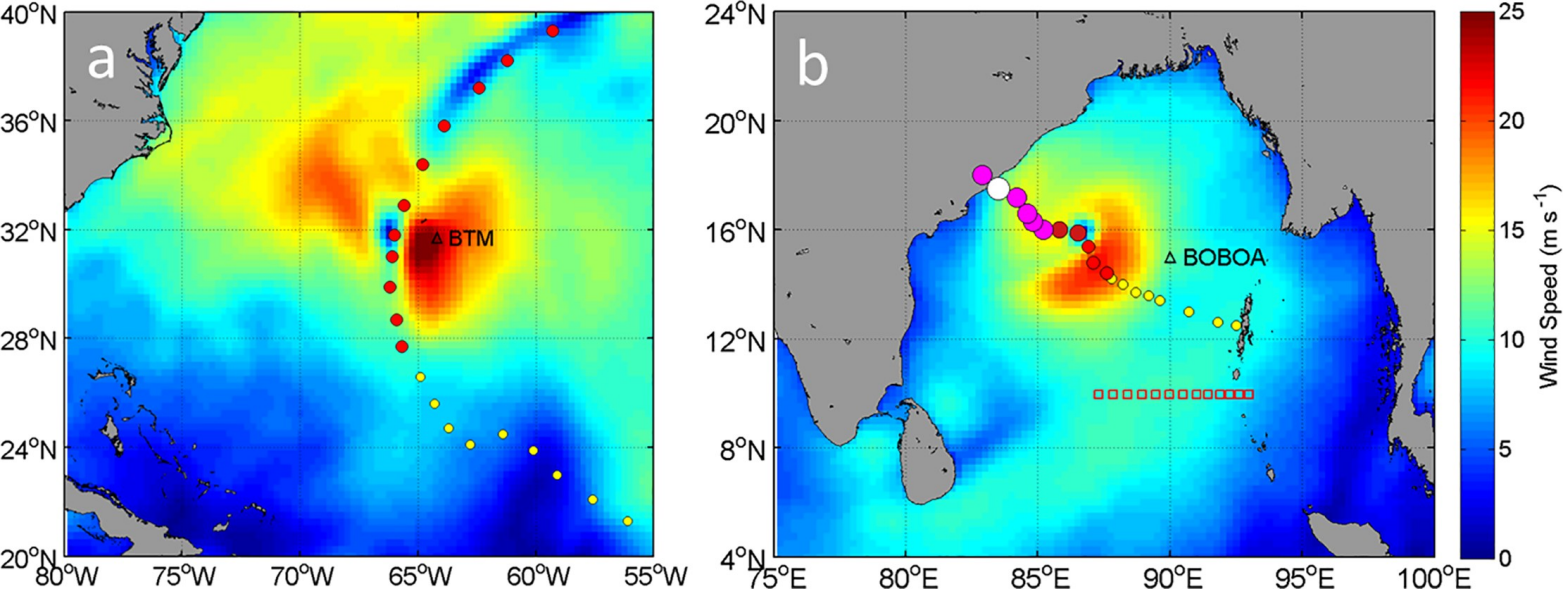
Research area. (a) The North Atlantic Ocean, track of tropical cyclone (TC) Florence, and the Bermuda Testbed Mooring (BTM) mooring at 64.2°W and 31.7°N (black triangle). (b) The Bay of Bengal (BoB), track of TC Hudhud, and the Bay of Bengal Ocean Acidification (BOBOA) mooring at 90°E and 15°N (black triangle). Shading in the Fig 1A and 1B shows wind speeds at 1200Z UTC on 11 September 2006 and 0600Z UTC on 10 October 2014, respectively. The TC intensity is marked by color dots (yellow: tropical storm; red: TC 1; pink: TC 2 and white: TC 3), and the size of the dots increases for colors from yellow to white. The red squares in Fig 1B show the World Ocean Circulation Experiment cruise sample stations which were conducted on October 1995.

Atmospheric pCO_2_ at ~ 1.5 m above the surface and surface seawater pCO_2_, SST and sea surface salinity (SSS) at ~ 0.5 m depth have been collected from two open ocean mooring buoys, the Bermuda Testbed Mooring (BTM) at 31.72°N, 64.19°W and Bay of Bengal Ocean Acidification (BOBOA) mooring at 15°N, 90°E (https://www.pmel.noaa.gov/) (Fig 1). BTM mooring is located ~80 km southeast of Bermuda in the North Atlantic which experiences five TCs each year [24]. BOBOA mooring is located in a region of the tropical BoB with strong ocean-atmosphere interactions that also experiences four TCs every year [25]. A Moored Autonomous pCO_2_ (MAPCO_2_) system was deployed on both buoys. Previous study described the MAPCO_2_ system, data reduction and processing and the uncertainty of each parameter, and provided a data set of 3-hourly pCO_2sea_, pCO_2air_, SST and SSS [4]. The total uncertainty of the MAPCO_2_ is < 2 μatm for pCO_2sea_ and < 1 μatm for pCO_2air_ [4].

In order to quantify the effects of TCs on the pCO_2sea_ and F_CO2_, the vertical profiles of in situ hydrographical parameters were examined. At the BTM mooring site, the vertical profiles of temperature, salinity, DIC, TA and Chla were observed from the Bermuda Atlantic Time-series Study (BATS) site (http://bats.bios.edu/) on 6 and 15 September 2006. Shipboard measurements of temperature, salinity, DIC, TA and Chla were collected at depths of about 3, 20, 40, 60, 80, 100, 120, 140, 160 and 200 m in the upper ocean on 6 September 2006. On 15 September 2006, measurements of temperature and salinity were collected at 3, 10, 20, 30, 50, 80, 103 and 122 m depths, while Chla was collected at 3, 30, 50, 80 103 and 122 m depths. At the BOBOA mooring site, the observations of hourly temperature and salinity data at depths of 1, 5, 10, 20, 40, 60, 80, 100, 120, 140, 160, 180 m were collected from the Research Moored Array for African–Asian–Australian Monsoon Analysis and Prediction (RAMA) in the BoB (https://www.pmel.noaa.gov/gtmba/) [26].

### Tropical cyclone and satellite data

The 6-hourly central locations of TCs were derived from the best-track data of the Joint Typhoon Warning Center (weather.unisys.com/hurricane/). During the mooring observation periods of October 2005–October 2007 at the BTM and November 2013–January 2017 at the BOBOA mooring site, only TC Florence and Hudhud respectively have passed the two moorings within 200 km. The center of TC Florence was about 165 km away from the BTM mooring site on 11 September 2006 (Fig 1A) and TC Hudhud was about 178 km to the south of the BOBOA mooring site on 9 October 2014 (Fig 1B).

Atmosphere and ocean surface conditions at the mooring buoys before, during and after the passage of TC Hudhud and Florence were determined from four different remote sensed products. The products include: the 6 hourly, 25 km, cross-calibrated multiplatform (CCMP) ocean surface winds from the National Aeronautics and Space Administration (NASA) [27]; the daily, 25 km, rainfall from the Tropical Rainfall Measuring Mission (https://disc2.gesdisc.eosdis.nasa.gov/data/TRMM_L3/); the daily, 1 km, NASA Multi-Sensor Merged Ultrahigh Resolution (MUR) SST (http://data.nodc.noaa.gov/ghrsst/L4/GLOB/JPL/); the daily, 4 km, Aqua Moderate Resolution Imaging Spectroradiometer (MODIS-Aqua) from the NASA Ocean Color (https://oceancolor.gsfc.nasa.gov/cgi/l3). These remote sensing data were used for analysis of pCO_2sea_ and F_CO2_ variation. MATLAB 2018a software with m_map package was used to process and visualize all the data in this study.

### Computational analysis

In the present study, the translation speeds of a TC were estimated from time-varying positions of its center. To define the initial upper ocean states, MLD and isothermal layer depth (ILD) of the water were investigated. The MLD is defined as the depth at which density exceeds 0.2 kg m^–3^ from its surface value [28]. The ILD is defined as the depth at which temperature decreases by 1°C from the SST [29]. To remove the temperature effect on the pCO_2sea_, temperature-normalized pCO_2sea_ (NpCO_2,Tmean_) is calculated as follows [30]:

$${NpCO}_{2,Tmean}={pCO}_{2sea}\times {exp}^{0.0423({SST}_{mean}-SST)}$$ (1)

where, SST_mean_ is 26.84°C and 28.86°C, the mean temperature during the study period, for TC Florence on 6–17 September 2006 and Hudhud on 4–15 October 2014, respectively.

The F_CO2_ (mmol CO_2_ m^–2^ d^–1^) is often calculated using the bulk equation as follows:

$$F_{CO2}=k\times K_{H}\times ({pCO}_{2sea}-pCO_{2air})$$ (2)

Where, k is the gas transfer velocity of CO_2_ (cm h^–1^), K_H_ is CO_2_ the solubility of surface seawater (mol L^–1^ atm^–1^) calculated according to Weiss (1974) [31]. The direction of F_CO2_ is determined by the partial pressure difference, pCO_2sea_−pCO_2air_ (ΔpCO_2_, μatm).

The transfer velocity, k, is frequently parameterized as a function of wind speed at 10 m height (u_10_, m s^–1^). The recently updated relationship [3] was used in this study to calculate k:

$$k=0.251{u_{10}}^{2}{\left(S_{c}/660\right)}^{-0.5}$$ (3)

where, S_c_ is the Schmidt number calculated according to well established methodology [3]. The total uncertainty of F_CO2_ is 20% which is determined mostly by the uncertainty of k [14].

To explore the effect of vertical mixing on the variation of pCO_2sea_ through these factors, the most widely used equation [23] were used:

$$\begin{array}{c} {dpCO}_{2}=({\partial pCO}_{2}/\partial T)dT+({\partial pCO}_{2}/\partial DIC)dDIC\\ +({\partial pCO}_{2}/\partial TA)dTA+({\partial pCO}_{2}/\partial S)dS \end{array}$$ (4)

where T and S denote SST and SSS respectively. The effects of each factor on the pCO_2sea_ can be determined from the seawater CO_2_ thermodynamics relationships as:

$$({\partial pCO}_{2}/\partial T)/{pCO}_{2}={0.0423℃}^{-1}$$ (5)

$$({\partial pCO}_{2}/\partial DIC)(DIC/{pCO}_{2})=8$$ (6)

$$({\partial pCO}_{2}/\partial TA)(TA/{pCO}_{2})=-7.4$$ (7)

$$\left(\partial {\mathrm{pCO}}_{2}/\partial S\right)\left(S/{\mathrm{pCO}}_{2}\right)=0.93$$ (8)

## Results

### Tropical Cyclone Florence

#### Observations of wind speed, SST, SSS, and pCO_2_

**Fig 2:**
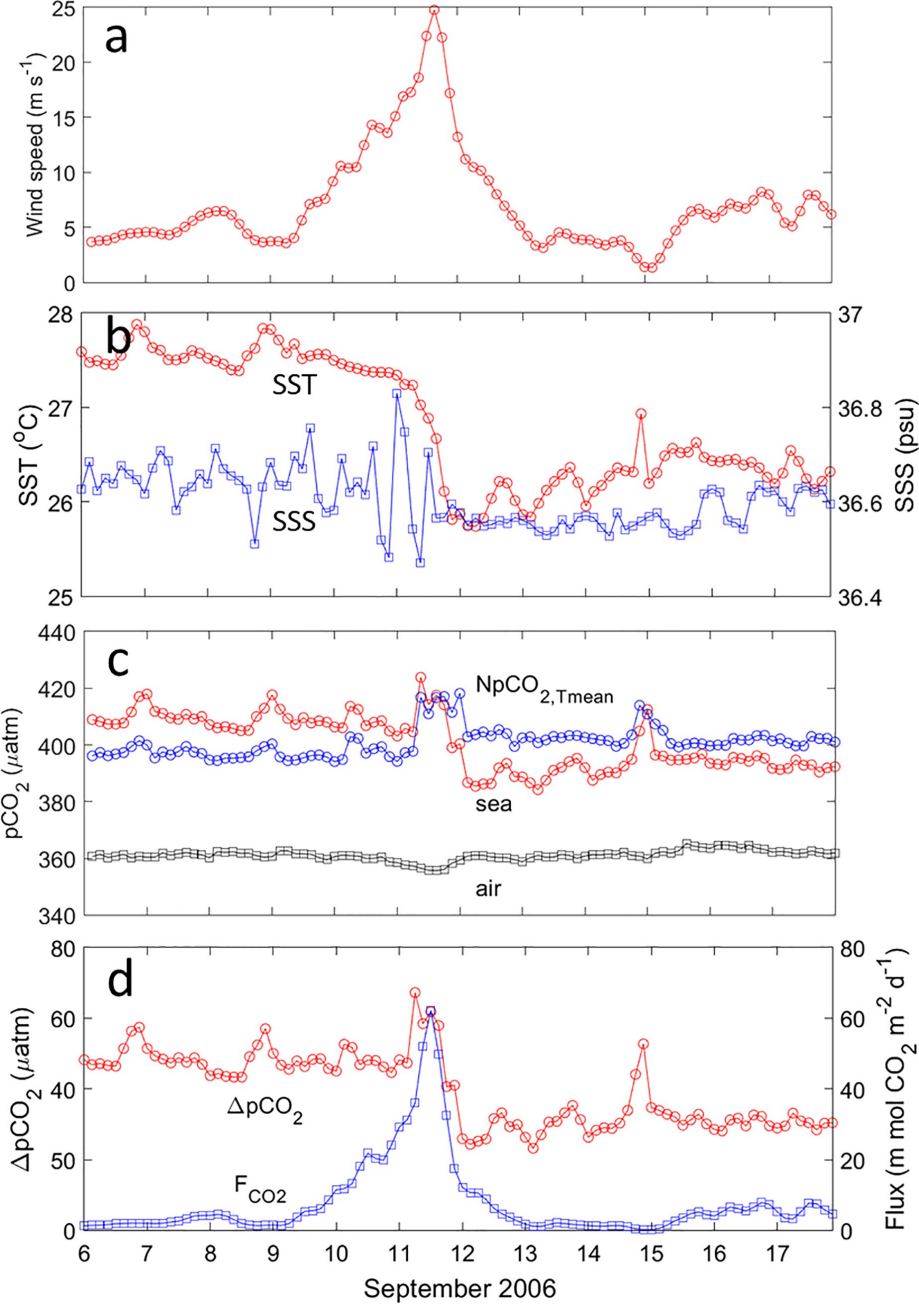
Three-hourly observations during Florence’s passage on 6–17 September 2006 at the BTM mooring site. (a) The CCMP wind speeds at 10 m height. (b) Sea surface temperature (SST) and sea surface salinity (SSS) at 0.5 m depth, (c) PCO_2air_ at 0.5 m height, pCO_2sea_ at 0.5 m depth and NpCO_2,Tmean_. (d) ΔpCO_2_ and F_CO2_.

Florence was a Category 1 cyclone according to the Saffir-Simpson hurricane scale (Fig 1A). It moved northwestward after its generation and then headed northeastward on 11 September 2006. The center of Florence was about 165 km to the west of the BTM mooring site at 1200Z UTC on 11 September 2006 with a translation speed of 7.23 m s^–1^. Before Florence’s passage on 6–9 September 2006, the CCMP wind speeds at the BTM mooring site were weak with the four-day averaged value of 5.00±1.29 m s^–1^ (one standard deviation, hereafter the same) (Fig 2A). The wind speeds sharply increased to 24.8 m s^–1^ on 11 September with the four-day averaged value of 11.53±5.75 m s^–1^ during Florence’s passage on 10–13 September. The wind speeds then gradually decreased to light wind (4.66±1.89 m s^–1^) after Florence’s passage on 14–17 September.

The SST at the BTM mooring site was usually high (> 27.5°C) with the four-day average value of 27.58±0.13°C before Florence’s passage (Fig 2B). Significant SST cooling was observed during (26.57±0.66°C) and after (26.37±0.18°C) Florence’s passage (Table 1). The SSS before, during and after the TC was low and constant (36.61±0.06 psu), except for minor fluctuation on 10–11 September which was mainly due to the precipitation and high wind speeds accompanied by the storm.

**Table 1:** Statistics of water properties, pCO_2_ and CO_2_ fluxes (mean±standard deviation) before, during and after the passages of tropical cyclone Florence at the BTM and Hudhud at the BOBOA mooring site.

	Date	SST (°C)	SSS (psu)	pCO_2sea_ (μatm)	pCO_2air_ (μatm)	F_CO2_ (mmol CO_2_ m^–2^ d^–1^)
**Hurricane Florence (September 2006)**						
**Before**	6–9 Sep	27.58±0.13	36.65±0.04	409.44±3.34	361.18±0.80	2.89±1.72
**During**	10–13 Sep	26.57±0.66	36.59±0.08	399.16±11.19	359.52±1.70	16.34±16.23
**After**	14–17 Sep	26.37±0.18	36.58±0.04	394.28±4.50	362.54±1.34	3.84±2.47
**Tropical Cyclone Hudhud (October 2014)**						
**Before**	4–7 Oct	29.35±0.17	32.27±0.08	379.71±3.93	371.62±2.39	0.19±0.21
**During**	8–11 Oct	28.60±0.27	32.84±0.43	388.12±7.94	374.32±7.52	4.76±2.72
**After**	12–15 Oct	28.64±0.28	33.16±0.09	394.52±5.83	375.10±1.53	1.15±1.34

The BTM moored buoy data revealed strong temporal variations in pCO_2sea_ (400.96±9.56 μatm) and weak temporal variations in pCO_2air_ (361.08±1.81 μatm) with the passage of TC Florence (Fig 2C). The pCO_2sea_ gradually decreased from 409.44±3.34 before to 399.16±11.19 μatm during and 394.28±4.50 μatm after Florence’s passage (Table 1). Similarly, the ΔpCO_2_ decreased with time (Fig 2D). The sudden rise in pCO_2sea_ on 11 September is consistent with the onset of entrainment into the mixed layer of water with high pCO_2sea_ [22]. The NpCO_2,Tmean_ (400.88±5.18 μatm) remained almost constant during the time period except for a sudden increase on 11 September. Slight decrease of NpCO_2,Tmean_ mainly due to the CO_2_ outgassing was observed during the Florence passage. After Florence’s passage, the NpCO_2,Tmean_ was about 5 μatm higher than before. This suggested that slightly high carbon-rich water were mixed to the surface due to TC-induced vertical mixing.

The positive ΔpCO_2_ over the entire observation time indicated CO_2_ flux from the ocean to the atmosphere. The averaged F_CO2_ of 2.89±1.72 mmol CO_2_ m^–2^ d^–1^ was observed before Florence’s passage (Fig 2D). Extremely high F_CO2_ (38.79 mmol CO_2_ m^–2^ d^–1^) was observed on 11 September due to the highest wind speeds. The F_CO2_ decreased abruptly to near zero as the wind speeds weakened to the pre-storm values. The F_CO2_ enhancement caused by Florence during the study period was about 57.62 ±11.52 mmol CO_2_ m^–2^.

#### Satellite–Derived precipitation, SST, and Chla

**Fig 3:**
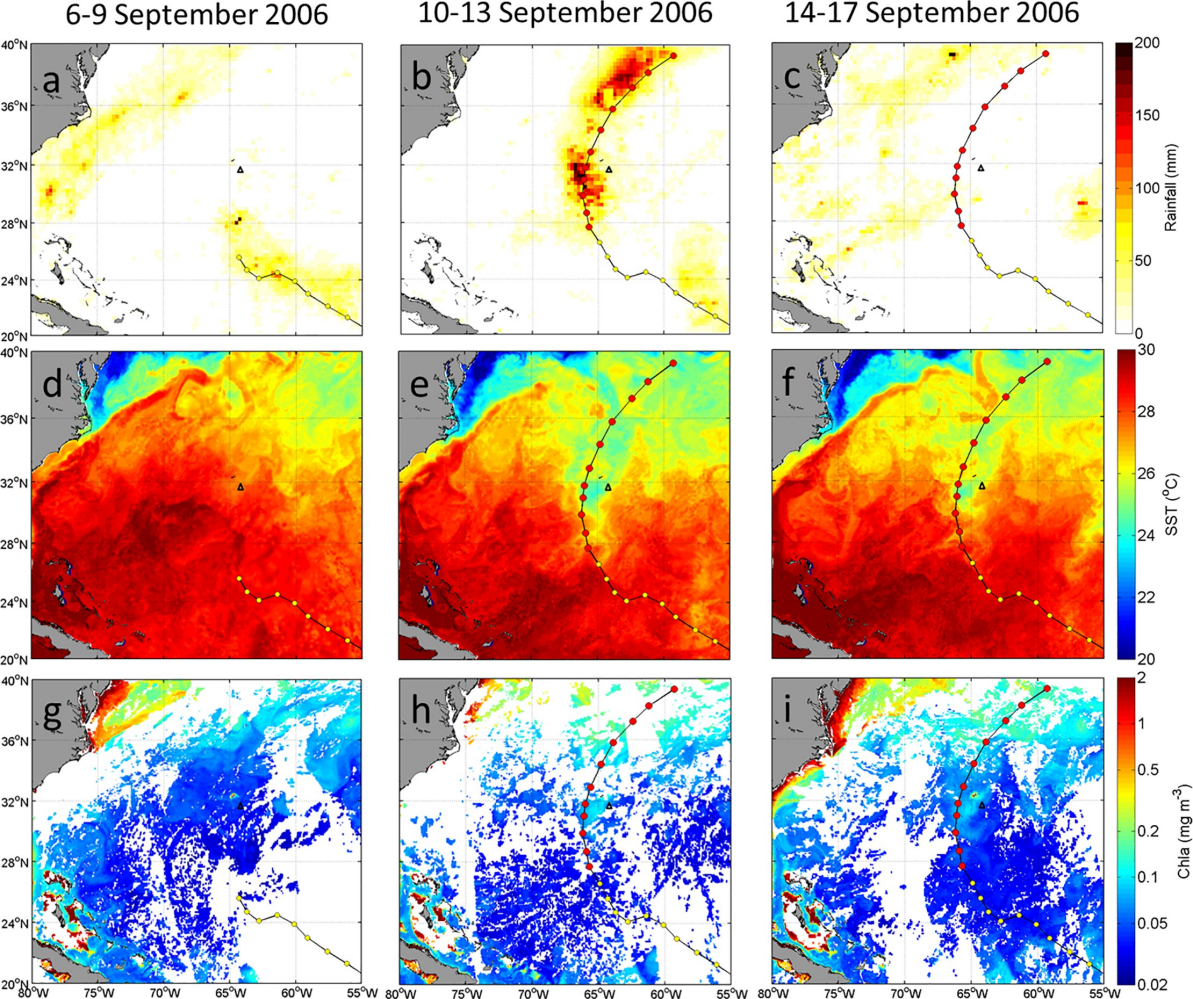
Satellite images of four-day averaged (a-c) precipitation (mm d^–1^); (d-f) sea surface temperature (SST,°C), and (g-i) surface chlorophyll a (Chla, mg m^–3^) before (left panel), during (middle panel) and after (right panel) Florence’s passage over the North Atlantic Ocean on 6–17 September 2006. Black triangles represent the BTM mooring site.

In addition to the strong wind speeds, Florence also brought a large amount of precipitation (150 mm d^–1^) along the storm path during the TC’s passage. Before Florence’s passage, there was scarce precipitation over the most of the North Atlantic Ocean (Fig 3A), except for intense precipitation (about 70 mm d^–1^) over the northwestern and southeastern area. At the BTM mooring site, the precipitation increased from almost zero before to 24.5 mm d^–1^ during Florence’s passage (Fig 3B), as reflected by the freshening of the sea surface water at ~ 0.5 m (Table 1). Although the precipitation showed spatial variability on 14–17 September, scarce precipitation occurred at the BTM mooring site (Fig 3C).

The MUR SST images showed sea surface cooling after Florence’s passage (Fig 3D, 3E and 3F). The North Atlantic Ocean was dominated by high SST water (>29°C) before Florence’s passage, except for the low SST (<25°C) over the north of the Gulf Stream. During and after Florence’s passage, a low SST patch (~25°C) appeared to the right side of TC’s path, especially near the Bermuda Island (Fig 3E and 3F). At the BTM mooring site, the SST value decreased from 28.9°C to 27.4°C, with a cooling of 1.5°C, which coincided well with in situ observations.

The sea surface MODIS-Aqua Chla concentrations were slightly enhanced during the Florence period. Significant low Chla concentration (< 0.05 mg m^–3^) occurred over the central North Atlantic Ocean and high Chla in the coastal and northern areas (Fig 3G). At the BTM mooring site, the Chla concentration increased from 0.04 mg m^–3^ before to 0.06 mg m^–3^ during and after Florence’s passage (Fig 3H and 3I). The high Chla concentration patch (~ 0.15 mg m^–3^) at 65°W and 31°N was very well matched with the SST cooling patch.

#### Vertical distributions of temperature, salinity, DIC, TA and Chla

**Fig 4:**
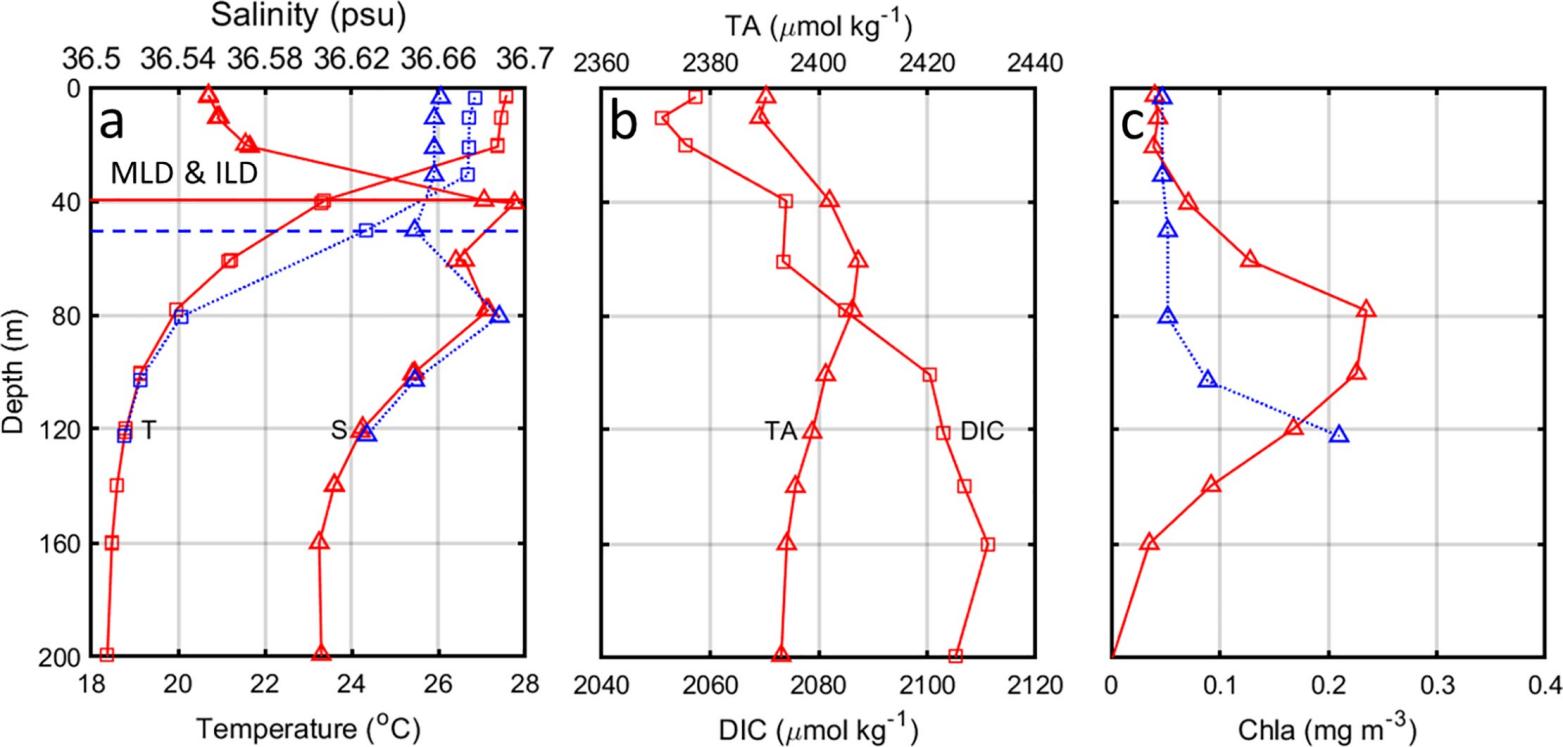
Vertical distributions of (a) temperature (squares) and salinity (triangles), (b) DIC (squares) and TA (triangles), and (c) Chla (triangles) before (red solid line) on 6 September 2006 and after (blue dashed line) Florence’s passage on 15 September 2006 based on two BATS cruises (http://bats.bios.edu/) which were quite close to the BTM mooring site.

Before Florence’s passage on 6 September 2006, a BATS cruise was conducted at 31.64°N, 64.19°E, quite close to the BTM mooring site. The vertical distributions of temperature and salinity indicated that the MLD and ILD were 39.7 m (Fig 4A, red line). Weak vertical salinity gradient of 0.003 psu m^–1^ was observed in the upper 39.7 m (note the small salinity scale in Fig 4A). After Florence’s passage on 15 September 2006, the MLD increased to 50.2 m based on another BATS cruise which was conducted at 31.67°N, 64.15°E. The deepened MLD suggested strong vertical mixing generated by Florence in the top 50.2 m depth. The observed temperature decrease in the top 30 m and increase in the subsurface layer at 30–60 m, combined with increased salinity in the top 20 m and decreased salinity at 20–60 m depth, suggested that the 39.7 m depth water was mixed to the surface due to the vertical mixing.

Prior to the storm on 6 September, the vertical profiles of DIC and TA indicated that the DIC (2054.6±3.2 μmol kg^–1^) and TA (2389.7±0.8 μmol kg^–1^) in the upper mixed layer of 39.7 m were almost constant (Fig 4B). The DIC linearly increased from 2057.3 μmol kg^–1^ at the sea surface to 2074 μmol kg^–1^ at 39.7 m depth. The TA increased from 2390.3 μmol kg^–1^ at the sea surface to 2402 μmol kg^–1^ at 39.7 m and linearly decreased to 2393.1 μmol kg^–1^ at 200 m after it reached maximum value of 2407.3 μmol kg^–1^ at 60.9 m depth. The vertical DIC gradient of 0.53 μmol kg^–1^ m^–1^ was higher than the vertical TA gradient of 0.34 μmol kg^–1^ m^–1^ in the mixed layer. Based on the MATLAB CO2SYS version 1.1 program [32], the pCO_2sea_ was about 423.56 μatm which was about 13 μatm higher than the in situ pCO_2sea_ of 410.74 μatm. This difference might be due to the depth of in situ pCO_2sea_ (0.5 m) that was shallower than that of calculated pCO_2sea_ (3 m). Unfortunately, DIC and TA were not measured in the cruise after Florence’s passage on 15 September.

Vertical profiles of Chla concentrations indicated that Chla concentration was low in the surface water at about 3 m depth (0.04 mg m^–3^) and in the top 40 m (< 0.07 mg m^–3^) before Florence’s passage on 6 September (Fig 4C). The weak subsurface chlorophyll maximum (0.235 mg m^–3^) was observed at 78 m depth. After Florence’s passage on 15 September, surface Chla concentration at about 3 m depth slightly increased to 0.047 mg m^–3^ which was matched well with the MODIS-Aqua result.

### Tropical Cyclone Hudhud

#### Observations of wind speed, SST, SSS, and pCO_2_

**Fig 5:**
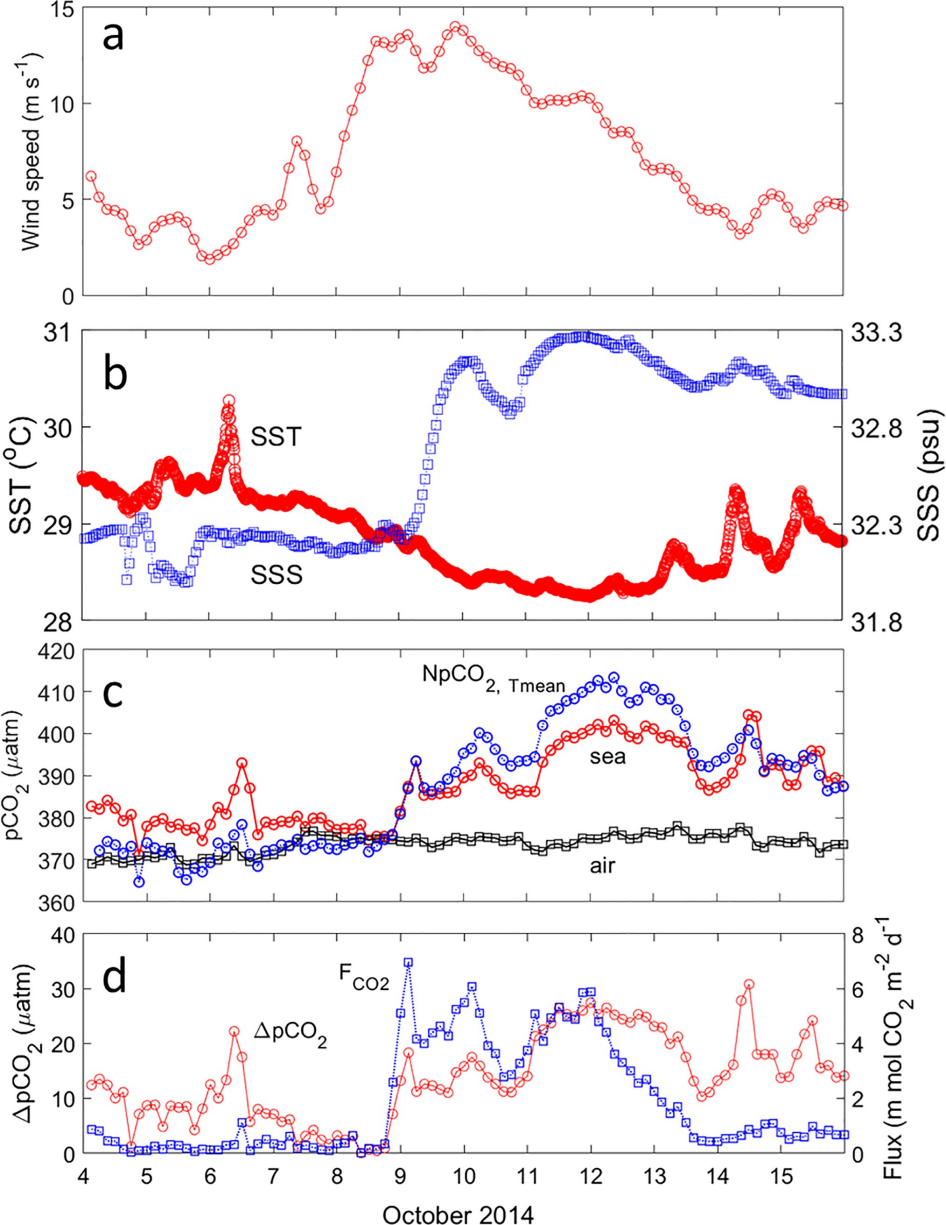
Three-hourly during the Hudhud’s passage on 4–15 October 2014 at the BOBOA mooring site. (a) The CCMP wind speeds at 10 m height. (b) Sea surface temperature (SST) and sea surface salinity (SSS) at 0.5 m depth. (c) PCO_2air_ at 0.5 m height, pCO_2sea_ at 0.5 m depth and NpCO_2,Tmean_. (d) ΔpCO_2_ and F_CO2_.

Hudhud was a Category 4 cyclone (Fig 1B). It was about 178 km to the BOBOA mooring site at 0000Z UTC on 9 October with a translation speed of 2.58 m s^–1^ and it moved northwestward before its landfall on 10 October 2014. Before Hudhud’s passage on 4–7 October 2014, the CCMP wind speeds at the BOBOA mooring site were weak with the four-day averaged value of 4.21 m s^–1^ (Fig 5A). The high wind speeds of 11.70 m s^–1^ were observed during Hudhud’s intrusion on 8–11 October. The wind speeds weakened to 5.64 m s^–1^ after Hudhud’s passage on 12–15 October.

Before Hudhud’s arrival, the BOBOA mooring site was characterized by warm SST (29.35±0.17°C) (Fig 5B). The SST decreased to 28.60±0.27°C and 28.64±0.28°C during and after Hudhud’s passage respectively, with maximum reduction (> 1°C) on 11–12 October (Table 1). Prior to Hudhud, the SSS was generally low (<32.4 psu) and remained constant (32.27±0.08 psu) (Fig 5B). The SSS rapidly increased to > 33 psu on 9 October at 1517 UTC, which suggested that subsurface salty waters were mixed or uplifted to the surface.

The pCO_2sea_ at the BOBOA mooring site showed strong temporal variation (387.45±8.60 μatm) during the study period (Fig 5C). Meanwhile, the pCO_2air_ showed weak temporal variation (373.68±2.27 μatm). Four days before TC Hudhud, the pCO_2sea_ ranged from 370 to 385 μatm with the exception of high pCO_2sea_ values ~ 390 μatm around noon local time on 6 October. The pCO_2sea_ increased to 388.12±7.94 μatm and 394.52±5.83 μatm during and after Hudhud’s passage, respectively. Similarly, the ΔpCO_2_ increased with time. Compared with TC Florence, the NpCO_2,Tmean_ changes during Hudhud’s passage showed different variation. It sharply increased from lower than the pCO_2sea_ before the Hudhud to about 9.5 μatm higher than the pCO_2sea_ on 11 October during the TC passage. After Hudhud's passage, the NpCO_2,Tmean_ was about 27.32 μatm higher than before. These changes suggested that carbon-rich waters were mixed to the surface. The NpCO_2,Tmean_ gradually decreased to the same value as the pCO_2sea_ after 14 October, suggesting an occurrence of biological DIC consumption.

Generally, the pCO_2sea_ was oversaturated with respect to the atmosphere with the averaged F_CO2_ value of 0.19±0.21 mmol CO_2_ m^–2^ d^–1^ before Hudhud’s passage (Fig 5D). The F_CO2_ suddenly increased to 5.26 mmol CO_2_ m^–2^ d^–1^ on 9 October due to the high wind speeds. The four-day averaged F_CO2_ gradually decreased from 4.76±2.72 mmol CO_2_ m^–2^ d^–1^ during to 1.15±1.34 mmol CO_2_ m^–2^ d^–1^ after the storm.

#### Satellite–Derived precipitation, SST, and Chla

**Fig 6:**
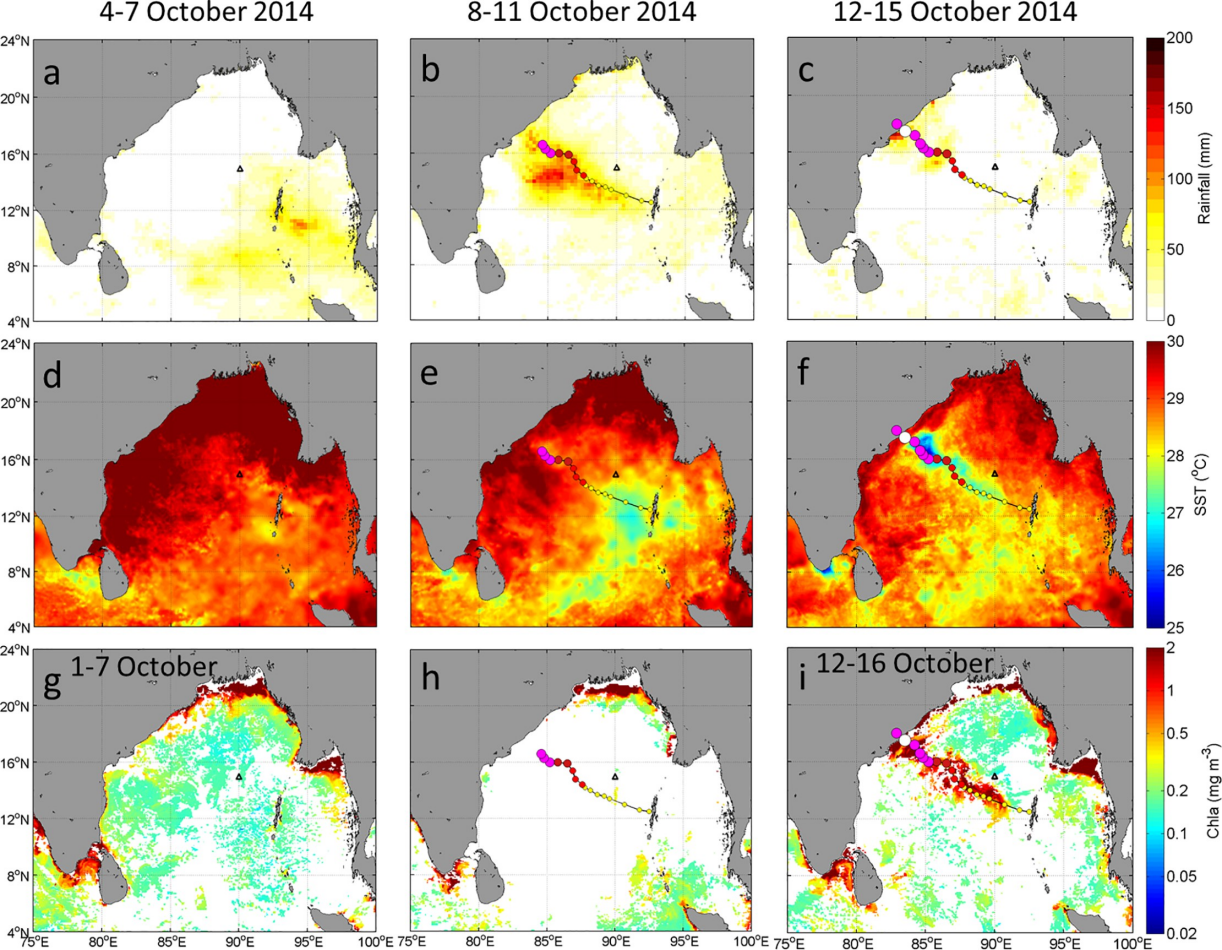
Satellite images of four-day averaged (a-c) precipitation (mm d^–1^); (d-f) sea surface temperature (SST,°C), and (g-i) surface chlorophyll a (Chla, mg m^–3^) before (left panel), during (middle panel) and after (right panel) Hudhud’s passage over the Bay of Bengal on 4–15 October 2014. Black triangles indicate the BOBOA mooring site. The Chla concentrations before, during and after Hudhud’s passage were averaged from 1–7, 8–11 and 12–16 October 2014, respectively.

Before Hudhud’s passage, scarce precipitation occurred over the most area of the BoB area (Fig 6A), except for some precipitation (about 50 mm d^–1^) over the southeastern area of the BoB. During the storm on 8–11 October, extremely heavy precipitation (150 mm d^–1^) along the storm path was brought by Hudhud (Fig 6B). At the BOBOA mooring site, the precipitation slightly increased from 8.5 mm d^–1^ before to 16.3 mm d^–1^ during Hudhud’s passage. There was scarce precipitation (< 1 mm d^–1^) after Hudhud’s passage (Fig 6C).

The SST averaged on 4–7 October suggested that high SST (> 29°C) prevailed in the whole BoB under the condition of no TC (Fig 6D). During Hudhud’s passage, the decreasing tendency of SST was evident in the TC generation area (Fig 6E). After Hudhud’s passage, a low SST patch appeared along the TC path centered at 86°E, 16°N (Fig 6F) under the maximum intensity and slow translation speed. The Hudhud-induced SST cooling was about 1°C at the BOBOA mooring site, which coincided well with in situ observations.

Before Hudhud’s passage, the seven-day averaged Chla concentrations showed low values (< 0.2 mg m^–3^) over the most BoB open ocean (Fig 6G). Unfortunately due to the cloud coverage during Hudhud’s passage, no valid data could be obtained to derive Chla images at the BOBOA mooring site (Fig 6H). The sea surface Chla concentrations were significantly enhanced (from < 0.2 to 0.5–4.0 mg m^–3^) after Hudhud’s passage along the storm path (Fig 6I). It should be noted that the patch of enhanced Chla concentration approximately matched with the low SST patch. At the BOBOA mooring site, the Chla concentration increased from 0.16 mg m^–3^ before Hudhud‘s passage on 7 October to 0.25 mg m^–3^ after Hudhud‘s passage on 14 October.

#### Vertical distributions of temperature and salinity

**Fig 7:**
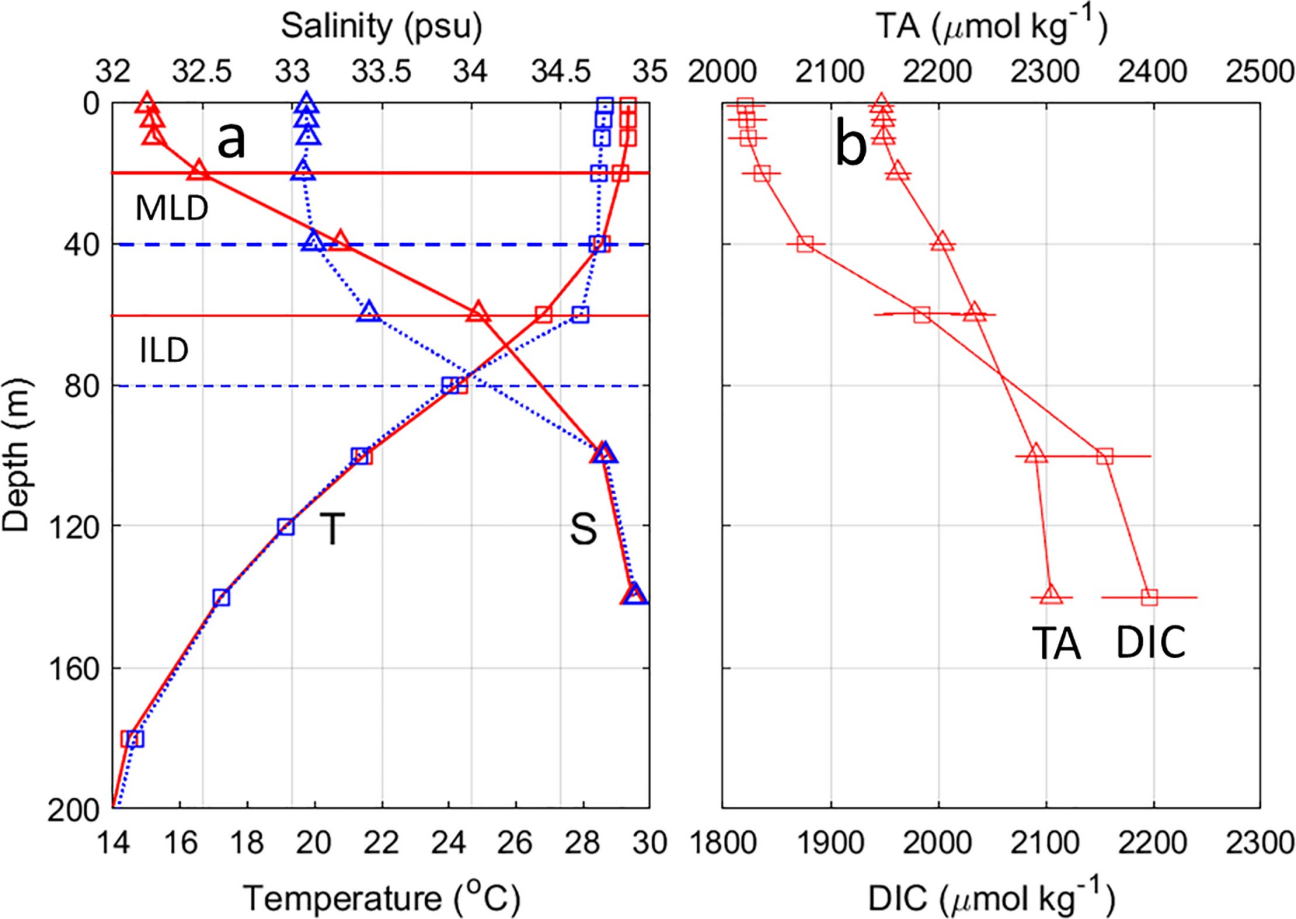
Vertical distributions of (a) four-day averaged temperature (squares) and salinity (triangles), and (b) DIC and TA before (4–7 October 2014, red solid line) and after (12–15 October 2014, blue dashed line) Hudhud’s passage at the BOBOA mooring site. The DIC and TA concentrations were derived from the DIC-salinity (DIC = 51.725 × salinity + 159.61, R^2^ = 0.959, *P*<0.001) and TA-salinity (TA = 52.558 × salinity + 454.16, R^2^ = 0.995, *P*<0.001) relationships in the upper layer of the ILD, and DIC-TA (DIC = 2.9649 × TA– 4638.7, R^2^ = 0.784, *P*<0.001) and TA-salinity relationships (TA = 82.862 × salinity– 587.86, R^2^ = 0.763, *P*<0.001) in the layer between ILD and the depth at which temperature decreases by 3°C from the SST.

The vertical distributions of four-day averaged (4–7, 8–11 and 12–15 October 2014) temperature and salinity indicated strong vertical mixing in the top 60 m (Fig 7A). The MLD was 20 m before the storm and increased to 40 m after the storm and the ILD deepened from 60 m to 80 m. The mean salinity in the top 40 m was increased from 32.58 psu before to 33.08 psu after Hudhud‘s passage. Strong vertical salinity gradient of 0.031 psu m^–1^ was observed in the upper 60 m. The decreased temperature in the top 40 m and increased temperature in the subsurface layer at 40–80 m, combined with the increased salinity in the top 38 m and decreased salinity at 38–100 m depth suggested that the 60 m depth water was mixed to the surface due to the vertical mixing.

## Discussion

The pCO_2sea_ is primarily a function of temperature, DIC, TA and salinity. DIC variability is controlled by vertical entrainment, phytoplankton photosynthesis, precipitation and air-sea CO_2_ exchange processes in the open ocean. Due to TC “wind-pump” effects, cold and CO_2_-rich subsurface waters are upwelled and mixed with the surface water, which in turn can decrease [5, 20–21] or increase [6–7] the surface pCO_2sea_, deepening on whether cooling or carbon enrichment dominates carbonate chemistry. Biological production dominated the decreasing pCO_2sea_ in the sea east of Sri Lanka during summer monsoon [33]. Furthermore, the sea surface chemical dilution by heavy precipitation can decrease the pCO_2sea_ [6, 34].

### Decreased pCO_2sea_ due to weak vertical salinity gradient

The four-day averaged pCO_2sea_ values at the BTM mooring site were lower during (10.28±11.68 μatm) and after (15.16±5.60 μatm) Florence’s passage than before the TC (409.44±3.34 μatm; Fig 2C). The F_CO2_ caused by Florence was 57.62±11.52 mmol CO_2_ m^–2^. Given the annual CO_2_ influx of about 815±251 to 1295±294 mmol CO_2_ m^–2^ in the North Atlantic near Bermuda [19], the impact of Florence’s passage on the local CO_2_ exchange was very significant. Based on the MODIS-Aqua images and in situ measurements, Chla concentration slightly increased from 0.04 mg m^–3^ before to 0.06 and to 0.06/0.047 mg m^–3^ during/after Florence’s passage, respectively (Figs 3G, 3H, 3I and 4C). This small increase in Chla concentration indicates a negligible effect of the phytoplankton photosynthesis on pCO_2sea_. The lack of biological drawdown was also manifested by the NpCO_2,Tmean_, which was always higher than the pCO_2sea_ after Florence’s passage.

The vertical mixing changed the temperature, DIC, TA and salinity in the surface water, which subsequently changed the pCO_2sea_. As shown in Figs 2B, 3D, 3E and 3F, Florence rapidly decreased the SST by 1.21±0.22°C. Using Eq (5), the SST cooling caused a decrease of 20.93±3.81 μatm on pCO_2sea_ (Table 2). Due to the weak vertical salinity gradient in the upper layer of the ILD (0.003 psu m^–1^) and moderate precipitation (24.5 mm d^–1^) on 10–13 September 2006 (Fig 3B), the effect of SSS on pCO_2sea_ was slight, causing a decrease of 0.73±0.62 μatm calculated by Eq (8).

**Table 2:** Summary of changes (mean±standard deviation) in pCO_2sea_ due to changes in SST, SSS, DIC, TA, F_CO2_ and dChla between before and after TC’s passage.

Tropical Cyclone	In-situ pCO_2sea_ change	dSST	dSSS	dDIC	dTA	dF_CO2_	dChla	Total^a^
**Florence**	–15.16±5.60	–20.93±3.81	–0.73±0.62	12.41	–5.06	–3.42±0.39	Negligible	–17.73±3.88
**Hudhud**	14.81±7.03	–11.65±5.30	9.75±1.31	81.39±3.87	–49.26±0.55	–1.69±0.24	Significant decrease	28.54±6.72

^a^The total effect is calculated from dSST, dSSS, dDIC, dTA and dF_CO2_.

The CO_2_ efflux takes away the gaseous CO_2_ from the ocean which decreases the concentration of H_2_CO_3_, ${HCO}_{3}^{-}$ and ${CO}_{3}^{2-}$ (DIC) through the thermodynamic equilibrium. The pCO_2sea_ variation due to the gas exchange of CO_2_ can be estimated by the change of DIC in the mixed layer and the sum of the aqueous CO_2_ and H_2_CO_3_ [14, 35]. Assuming the MLD of 50.2 m after TC Florence, the pCO_2sea_ variation induced by the F_CO2_ is about –3.42±0.39 μatm (Table 2), which is consistent with previous studies [36–37].

The changes of DIC and TA concentration were derived from two BATS cruises which were conducted around the BTM mooring site. Considering the precipitation of about 98 mm (4 day×24.5 mm d^–1^) during the storm and the MLD of 50.2 m after the storm, the SSS after the storm of 36.58 psu on 14–17 September (Table 1) equals to the value of 36.65 psu without the precipitation. The estimated SSS of 36.65 psu coincided with the actual SSS of 36.66 psu on 15 September (Fig 4A). Given the salinity of 36.56 psu in the mixed layer and 36.68 psu at 39.7 m depth before the storm on 6 September, three parts of the 39.7 m depth water were mixed with one part of the surface mixed layer water (assuming horizontal processes are negligible). Consequently, the concentration of DIC and TA at the sea surface would have been increased from 2057.3 and 2390.3 μmol kg^–1^ to 2065.1 and 2394.3 μmol kg^–1^, respectively. Using Eqs (6) and (7), the increased DIC and TA lead to 12.41 and –5.06 μatm changes in pCO_2sea_, respectively. These changes are smaller than those induced by cooling (–20.93±3.81 μatm), but larger than those due to SSS dilution (–0.73±0.62μatm) and F_CO2_ changes (–3.42±0.39 μatm). The total decrease of pCO_2sea_ generated by TC Florence is estimated to be about 17.73±3.88 μatm (Table 2). This estimated decrease is comparable to the observed decrease in pCO_2sea_ by about 15.16±5.60 μatm. The ratio of DIC to TA (DIC/TA) slightly increased from 0.860 in the mixed layer to 0.863 below the ILD layer. The small DIC/TA difference corresponds to a pCO_2sea_ decrease of 15.16±5.60 μatm which agrees well with our previous study [14].

**Fig 8:**
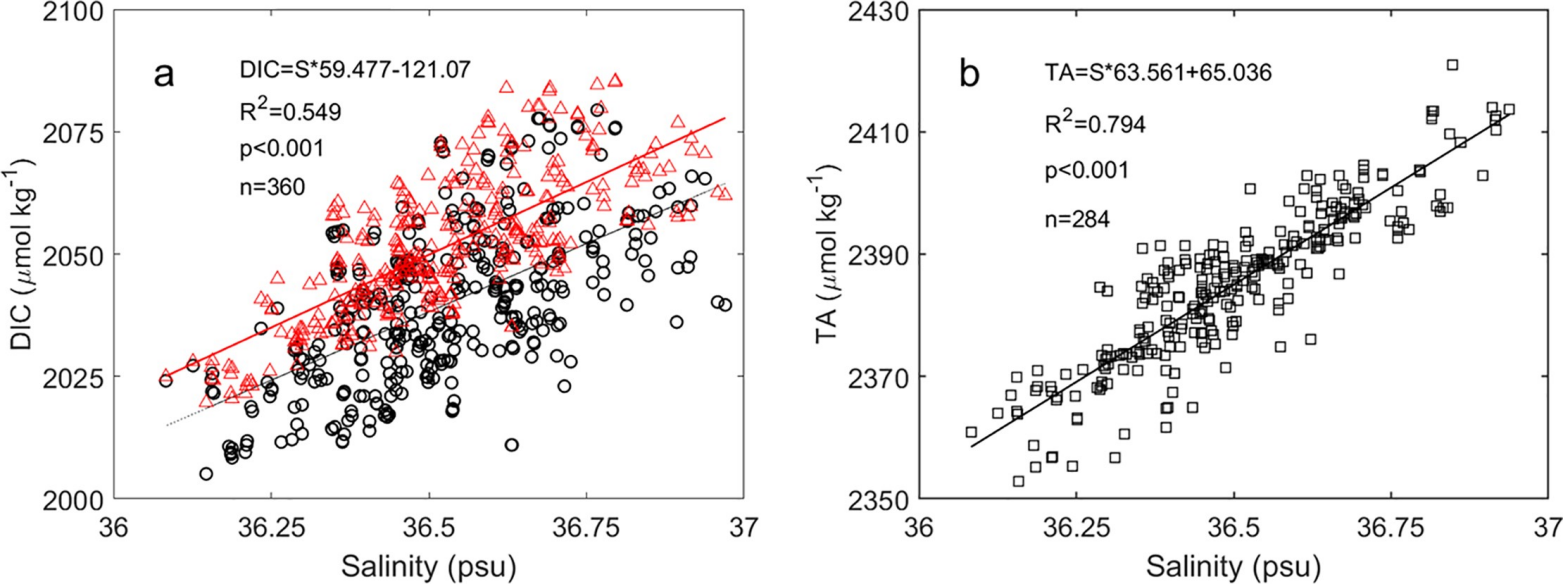
Linear regressions among DIC, TA and salinity around the BTM mooring site. (a) DIC and salinity. (b) TA and salinity. The black circles and red triangles in Fig 8A represent original DIC values and DIC adjusted to the year 2016 using the long-term increasing nDIC rate (0.86±0.11 μmol kg^–1^ year^–1^). The data were collected from the upper layer of the isothermal layer depth at the Bermuda Atlantic Time-series Study (BATS) site (http://bats.bios.edu/).

Regional regressions of DIC-salinity and TA-salinity were derived with the BATS bottle samples. For the upper layer of the ILD during the hurricane season on June-October [38], a moderate significant relationship between DIC and salinity based on 360 samples was derived (DIC = salinity × 55.8 + 0.5; R^2^ = 0.37, P<0.001) (Fig 8A). After adjusting DIC to the year 2016 using the long-term increasing nDIC (normalized to a salinity of 36.6) rate of 0.86±0.11 μmol kg^–1^ year^–1^ [39], a significant relationship between DIC and salinity was observed (DIC = salinity × 59.477–121.07 (±9.38); R^2^ = 0.549, P<0.001). Similarly, the TA-salinity relationship based on 284 samples yielded a statistically significant regression (TA = salinity × 63.561 + 65.036 (±5.66); R^2^ = 0.794, P<0.001) (Fig 8B). Highly correlated relationships were also observed between TA and salinity in the mixed layer and below mixed layer at the BATS site over a small salinity range of 36.2–36.9 [40]. As the DIC and TA were highly correlated with salinity, the weak vertical gradient in DIC and TA was mainly due to the weak vertical salinity gradient.

### Enhanced pCO_2sea_ due to strong vertical salinity gradient

After Hudhud’s passage, the four-day averaged pCO_2sea_ value at the BOBOA mooring site was about 14.81±7.03 μatm higher than before the storm (Fig 5C). As part of the upper 60 m waters were uplifted to the surface at the BOBOA mooring site where the subsurface chlorophyll maximum (~ 2 mg m^–3^) was located at about 45–55 m before the TC [9], high Chla concentration occurred in the sea surface waters. The increase of Chla concentration was mainly due to the upwelling (positive Ekman pumping velocity of Fig 4A in Xu et al, (2019) [11]) of high phytoplankton concentrations from the subsurface water to the surface [17]. The strong increase of Chla concentration is indicative of significant biological production, which results in pCO_2sea_ decrease in this case. The biological production also manifests itself by the NpCO_2,Tmean_ which was gradually decreased from 410.52 μatm on 11 October to 390.54 μatm on 15 October (Fig 5C).

As shown in Figs 5B, 6D, 6E and 6F, Hudhud quickly decreased the SST by 0.71±0.33°C. Using Eq (5), the SST cooling caused a change of –11.65±5.30 μatm in pCO_2sea_. Due to the strong vertical salinity gradient in the upper layer of the ILD (0.031 psu m^–1^), the SSS significantly increased to 33.16±0.09 psu, which caused an increase of 9.75±1.31 μatm in pCO_2sea_ (Table 2).

The F_CO2_ enhanced by TC Hudhud was estimated to be about 22.15±4.43 mmol CO_2_ m^–2^, which was higher than the value of 18.49±3.70 mmol CO_2_ m^–2^ estimated by previous study [14]. This difference was due to RAMA in situ winds used in the previous study [14], while CCMP winds were used in this study. Considering the annual F_CO2_ of 55.78±11.16 mmol CO_2_ m^–2^ for the BOBOA water [14], the impact of Hudhud’s passage on the local CO_2_ exchange was very significant. Assuming the MLD of 40 m after TC Hudhud, the effect of air-sea CO_2_ fluxes on the pCO_2sea_ variation is about –1.69±0.24 μatm.

**Fig 9:**
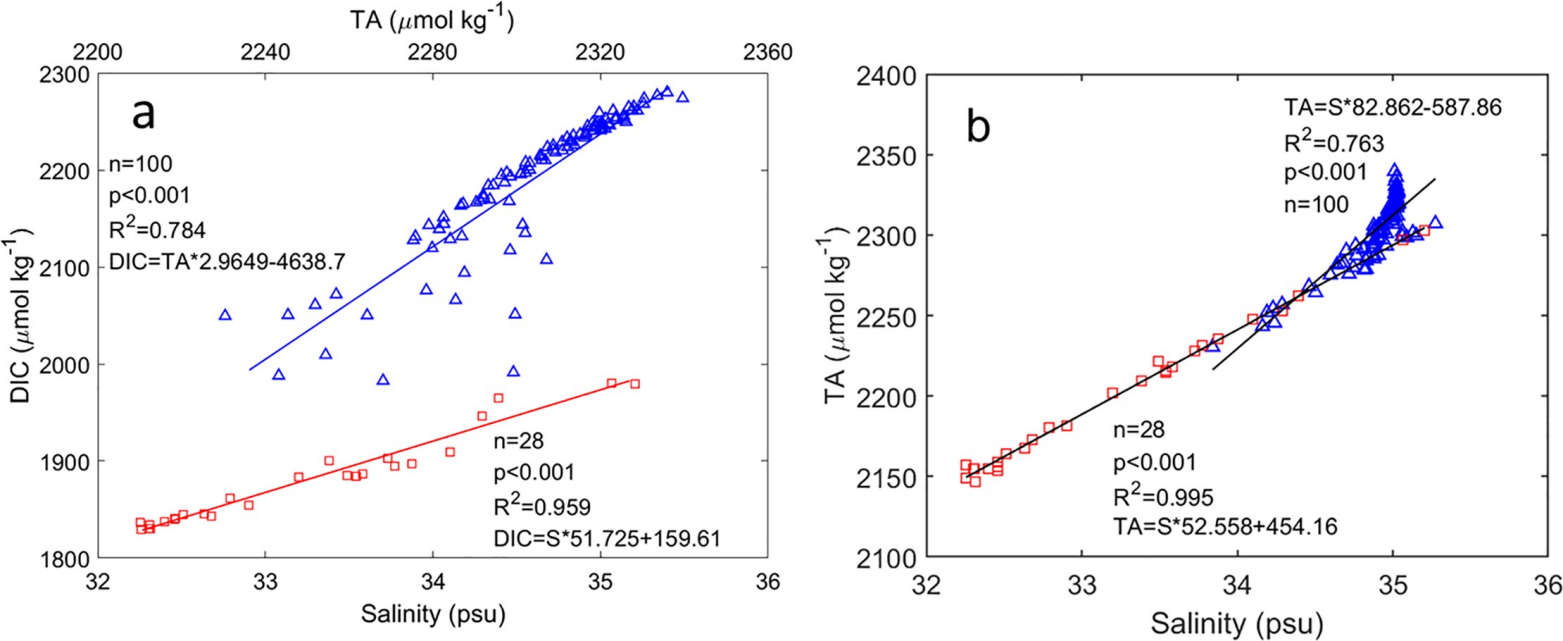
Linear regressions among DIC, TA and salinity around the BOBOA mooring site. (a) DIC and salinity, DIC and TA. (b) TA and salinity. Red squares and blue triangles represent samples collected from the upper layer of the isothermal layer depth (ILD) and the layer between ILD and 500 m depth, respectively. Samples were collected from the red squares as shown in Fig 1B. The data were collected during a WOCE cruise in October 1995 (http://cchdo.ucsd.edu/).

To estimate the effects of DIC and TA changes on the pCO_2sea_ variation at the BOBOA mooring site, regional relationships between salinity and DIC and TA were derived using a World Ocean Circulation Experiment (WOCE) cruise samples (http://cchdo.ucsd.edu/). Significant relationships between salinity and DIC (DIC = salinity × 51.725 + 159.61 (±9.25); R^2^ = 0.959, P<0.001) and TA (TA = Salinity × 52.558 + 454.16 (±3.19), R^2^ = 0.995, P<0.001) were derived in the upper layer of ILD between 10–18°N and 87–93°E (Fig 9). In the layer between ILD and 500 m, the significant relationship between salinity and TA (TA = 82.862 × Salinity − 587.86 (±10.56), R^2^ = 0.763, P<0.001), and significant relationship between TA and DIC (DIC = 2.9649 × TA– 4638.7 (±35.30), R^2^ = 0.784, P<0.001) were also derived. Using these relationships (shown also in Fig 9) and considering that salinity increased from 32.19 psu at the sea surface to 34.04 psu at 60 m depth, DIC was estimated to be about 1824.64±9.25 and 1981.21±35.30 μmol kg^–1^ and the TA, about 2146.00±3.19 and 2232.76±10.56 μmol kg^–1^ before the storm (Fig 7B). Strong vertical DIC and TA gradients of 2.61 μmol kg^–1^ m^–1^ and 1.45 μmol kg^–1^ m^–1^ in the upper layer of the ILD were observed, respectively.

As TC Hudhud mixed well the water in the top 60 m, DIC and TA in the sea surface would increase to 1873.49±36.49 and 2183.59±11.03 μmol kg^–1^, respectively. The increased DIC and TA lead to 81.39±3.87 and –49.26±0.55 μatm changes in pCO_2sea_, respectively. Therefore, the total effects of DIC and TA on the pCO_2sea_ enhancement were about 32.13±3.91 μatm, which was larger than the effect of SST on the pCO_2sea_ decrease 11.65±5.30 μatm. The total increase of pCO_2sea_ generated by Hudhud is estimated to be about 28.54±6.72 μatm (Table 2). Considering the phytoplankton photosynthesis processes may decrease the pCO_2sea_ by 9.5 μatm which was estimated from the NpCO_2,Tmean_, this likely leads to the observed increase in pCO_2sea_ of 14.81±7.03 μatm.

At the BTM mooring site, the decreased pCO_2sea_ after TC Florence was due to the weak vertical salinity gradient in the upper layer of the ILD (0.003 psu m^–1^). The effects of DIC and TA on the pCO_2sea_ enhancement were far lower than the effect of temperature on the pCO_2sea_ decrease. At the BOBOA mooring site, the increased pCO_2sea_ after TC Hudhud was mainly due to the strong vertical salinity gradient in the upper layer of the ILD (0.031 psu m^–1^) that determined the supply of much salinity, DIC and TA from the thermocline. This salinity related increase in pCO_2sea_ basically compensated for the contribution of SST decrease. As both DIC and TA are strongly correlated with salinity, and the strong (weak) vertical gradient in salinity is accompanied by the strong (weak) vertical gradients in DIC and TA, we propose that the magnitude of the vertical salinity gradient in the upper layer is a good indicator of the pCO_2sea_ variation after the passage of TC. Compared with the explanation of pCO_2sea_ variation after TC’s passage by vertical differences in DIC/TA in the upper layer [14], our new approach is simpler and more accessible because salinity is easier to obtain than DIC and TA. Other studies support our hypothesis. Under the impact of typhoon Wutip, significant increase of pCO_2sea_ (~ 20 μatm) was observed in the South China Sea where the vertical salinity gradient in the upper layer of the ILD (~0.03 psu m^–1^) was strong (Fig 6B in Ye et al, (2017) [7]). Meanwhile, decreased pCO_2sea_ (25.6 μatm) after Hurricane Frances was reported in the Atlantic Ocean at 66°W, 22.21°N where the vertical salinity gradient in the upper layer of the ILD (< 0.01 psu m^–1^) was weak (Fig 2B in Huang and Imberger, (2010) [21]).

## Conclusions

The present study examined various contributing factors that induced the change of pCO_2sea_ following TCs “wind-pump” over the North Atlantic Ocean (Florence, 3–12 September 2006) and BoB (Hudhud, 8–11 October 2014) using mooring buoy measurements. Significantly decreased pCO_2sea_ after Florence’s passage and increased pCO_2sea_ after Hudhud’s passage were observed. The analysis showed that the waters below the ILD were uplifted to the surface through TC-“wind-pump” induced vertical mixing in both cases. The impacts of phytoplankton photosynthesis processes on the pCO_2sea_ variation depended on the intensity and depth of the subsurface chlorophyll maximum, and the impacts of air-sea CO_2_ fluxes on the pCO_2sea_ variation were relatively low. In situ measurements showed that DIC and TA are strongly correlated with salinity in the upper layer of the ILD during the tropical cyclone season in the North Atlantic Ocean and BoB. The magnitude of the vertical gradient in DIC and TA can be estimated by the magnitude of the vertical salinity gradient. The decreased pCO_2sea_ was mainly due to the weak vertical salinity gradient in the upper layer of the ILD. The contribution of the temperature cooling on the pCO_2sea_ decrease exceeded the joint contribution of the DIC and TA enhancement on the pCO_2sea_ increase. While the increased pCO_2sea_ was mainly due to the strong vertical salinity gradient in the upper layer of the ILD that supply much salinity, DIC and TA, which together exceeded the contribution of the temperature decrease. Therefore, in addition to the TC intensity and translation speed, the responses of pCO_2sea_ to TCs are different due to the preexisting upper ocean states, including the vertical profiles of DIC, TA, temperature and salinity. This study highlights that the magnitude of the vertical salinity gradient in the upper layer of the ILD is a good quantitative indicator in controlling the pCO_2sea_ variation.
